# Homebound Risk and Safety Hazards in Older Adults With Vision Impairment

**DOI:** 10.1093/geroni/igaf122.3409

**Published:** 2025-12-31

**Authors:** Jonathan Thomas, Xindi Chen, Louay Almidani, Seema Banerjee, Zhuochen Yuan, Aleksandra Mihailovic, Pradeep Ramulu

**Affiliations:** Texas A&M University Health Science Center, Bryan, Texas, United States; Johns Hopkins University School of Medicine, Baltimore, Maryland, United States; Johns Hopkins University School of Medicine, Baltimore, Maryland, United States; Johns Hopkins University School of Medicine, Baltimore, Maryland, United States; Johns Hopkins University School of Medicine, Baltimore, Maryland, United States; Johns Hopkins University School of Medicine, Baltimore, Maryland, United States; Johns Hopkins University School of Medicine, Baltimore, Maryland, United States

## Abstract

Homebound status is associated with multiple risk factors and worse health outcomes. However, limited knowledge exists about the association between vision impairment (VI) and becoming homebound. Therefore, this study examined the associations between VI and homebound status, utilization of home-based care, presence of tripping hazards, and likelihood of transitioning to homebound status in United States (US) older adults. Longitudinal data was collected from the 2021-2023 National Health and Aging Trends Study and included Medicare beneficiaries ≥71 years. The main exposure was any VI, defined as impairment in either distance (DVA) or near (NVA) visual acuity (>0.3 logMAR), or contrast sensitivity (CS;< 1.55 logCS). Outcomes included homebound status, presence of safety hazards, and utilization of home-based care. 3,002 participants (mean [SD] age, 78.8 [5.6] years; 55.3% female; 81.8% non-Hispanic White) were included in our study. The prevalence of being homebound was higher among those with any VI (9.2%,95%CI:7.3-11.5%) compared to no VI (4.0%,95%CI:3.0-5.3%). In multivariable logistic regression models, individuals with any VI had a significantly higher risk of becoming homebound or semi-homebound (HR = 1.35,95%CI:1.04-1.74,p=.02) compared to peers without any VI. Likewise, participants with any VI had a greater likelihood of having home hazards (OR = 1.88,95%CI:1.32-2.69, p=.001) and utilizing home-based care (OR = 1.31,95%CI:1.06-1.62,p=.01) compared to those without any VI. In conclusion, older US adults with VI were more often homebound, more likely to become homebound, had more home hazards, and more often utilized home-based care, highlighting the need to holistically evaluate a patient when determining the need for home care and tailoring interventions to maximize independence.

